# Evaluating trends in recruitment challenges in vape shop research, e-cigarette product characteristics and use among shop customers from 2019 to 2023: A mixed-methods study

**DOI:** 10.18332/tid/175729

**Published:** 2024-05-23

**Authors:** Jimi Huh, Artur Galimov, Leah Meza, Ellen Galstyan, Jennifer B. Unger, Lourdes Baezconde-Garbanati, Steven Sussman

**Affiliations:** 1 Department of Population and Public Health Sciences, Keck School of Medicine, University of Southern California, Los Angeles, United States

**Keywords:** policies, vape shops, e-cigarette products

## Abstract

**INTRODUCTION:**

Brick-and-mortar vape shops specialize in the sale of e-cigarettes and remain a primary source for purchasing emerging e-cigarette products. New regulatory policies have been implemented at local-, state- and federal-level; the retail environment at vape shops and product preferences among vape shop customers shifted accordingly.

**METHODS:**

From 2019 to 2023, we collected anonymous interview data from vape shop customers (n=572) from 83 vape shops in Southern California. We aggregated the data by month and treated each month as the unit of analysis to document changes in recruitment efforts among the vape shops in relation to major policy implementations over 4 years. We also examined the systematic fluctuations and trends in customers’ e-cigarette product preferences and nicotine content in these products.

**RESULTS:**

The monthly average shop-level consent rate was 52.9% (SD=8.7), with an overall decreasing trend over time. It was necessary for our data collection team to approach a greater number of vape shops to obtain consent with implementation of various state and federal tobacco regulations and following COVID-19. We observed an increase in the purchase of disposable products and nicotine concentrations in the products, while the average use frequency remained the same.

**CONCLUSIONS:**

Our findings demonstrated that user preferences, product characteristics and challenges in research involving vape shops are closely associated to changes in regulations. We documented a dramatic increase in nicotine concentration in products. Future policies restricting the amount of nicotine in tobacco products at the federal level are necessary to protect consumers from further nicotine addiction. This study provides documentation over time of the drastic increases in nicotine concentration among e-cigarette users as a result of the fluctuations in the product market. Regulating nicotine content in tobacco products could safeguard against further unsafe modifications in e-cigarettes and other types of tobacco products.

## INTRODUCTION

The vape shop retail environment has rapidly evolved and shifted with changes in e-cigarette policies and regulations^1^. Marketing strategies of manufacturers and retailers have swiftly responded to the ever-changing consumer landscape – including varying e-cigarette consumer demand throughout the COVID-19 pandemic – and the changing of federal and state tobacco regulations^2-4^. Brick-and-mortar vape shop retailers specialize in the sale and promotion of e-cigarettes and e-liquids, and remain one of the primary sources to purchase vaping products^5,6^. Further, vape shop employees serve as one of the main points of contact for those interested in trying e-cigarettes for the first time^7^.

### The evolution of e-cigarette products and contained nicotine

E-cigarette products have proliferated since the mid 2000s^8^, and are available in a variety of flavors and nicotine concentrations^9^. Vaping devices have also undergone substantial transformations from thin, disposable cigarette-resembling, ‘1st-generation’ devices to the newest modern-looking, closed-system disposable pod devices^2^. First-generation devices were designed to mimic traditional cigarettes, making them attractive to current smokers. These then evolved into the second-generation tubular-shaped, larger tank style, refill-able and rechargeable devices. Modular (box) products gained popularity for several years (the third generation) until JUUL introduced the fourth-generation pod-style devices in 2015^10^. The more recent pod products are smaller than previous (box) mod devices (thus offering concealability) and are intended for immediate use. Further, closed-system disposable pod devices (e.g. ‘Flum’) offer a sleek design with an extensive range of e-juice flavors and high nicotine concentration levels ranging 20–70 mg/mL^10-12^.

### Overview of federal- and state-wide e-cigarette product regulations from 2016 to 2022

**Figure 1:**
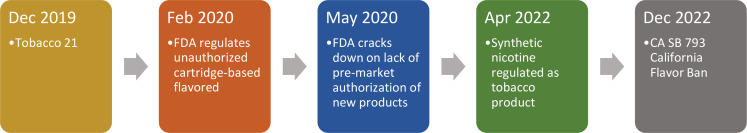
Federal and state-wide e-cigarette regulations during 2019–2023

The US Food and Drug Administration (FDA) established regulatory authority over e-cigarettes in 2016 because of their classification as tobacco products^1^. Since then, the federal government has implemented additional regulations affecting e-cigarette products and retail practices (e.g. the purchasing age of tobacco products was raised to 21 years of age)^13^. In February 2020, the US FDA enforced a regulation governing the manufacturing, distribution, marketing, and sale of prefilled cartridge-based flavored e-cigarettes, with the exemption of tobacco and menthol flavors^14,15^. In July 2020, the US FDA requested that 10 prominent e-cigarette manufacturers withdraw their flavored disposable e-cigarette products from the market due to the absence of the necessary premarket authorization^16^. Still, with ample availability, low pricing, and improved features [e.g. rechargeable battery technology, higher number of puffs (up to 7000) per device], novel vaping devices are easily accessible and continue to gain popularity (Figure 1).

In addition to the evolution of vaping devices, notable modifications in the nicotine formulation and type within e-cigarette products have been observed. Salt-based nicotine was introduced in 2015 and made popular in pod-style devices^17^. Synthetic nicotine e-cigarettes have emerged as an alternative to tobacco-derived nicotine vaping products and are marketed as containing ‘tobacco-free nicotine’, adding confusion and dubious claims of reduced risk in such products^18^. In response to the increased use of synthetic nicotine, US Congress passed a law that went into effect in April 2022, updating the Tobacco Control Act to include synthetic nicotine as a tobacco product and granting the FDA authority to regulate tobacco products containing nicotine from ‘any source’^19^.

In November 2022, California approved Proposition 31, a referendum to the 2020 California Senate Bill (SB) 793 that prohibits the retail sale of flavored tobacco products. Flavored tobacco products are defined in the bill as ‘any tobacco product that imparts a characterizing flavor other than tobacco’ (including menthol)^20^. As of 21 December 2022, e-cigarette retailers in California are now required to stop selling, offering for sale, and possessing with the intent to sell, flavored tobacco products^21^.

### Changes in vape shop retail environment

With the newly implemented federal- and state-wide regulations during this period, substantial changes have taken place in the e-cigarette market, including the type of e-cigarette products sold, and in the way vape shops operate. There are little data on how these changes in the tobacco product landscape (e.g. the evolution of devices and nicotine content) are associated with changes in vape shop customers’ product preferences (e.g. device type, flavor, nicotine level). Our research team has been in the unique position to observe these changes over time at brick- and-mortar retailers, as we have built rapport and collected data in the tobacco retail environment since 2014^2,22-27^. Along with the policy changes, our data collection team has encountered levels of enthusiasm, expressed by vape shop owners and employees in participating in research projects, which waxed and waned over the years, with an overall decreasing trend. We are one of the very few research teams in the forefront of monitoring and tracking the vaping industry since the early days of its inception, prior to the changes in regulation.

### Aims and hypotheses

Given our research team’s vantage point in conducting vape shop research over the past 9 years, we aim to document changes (successes and challenges) in recruitment efforts among the vape shop retailer environment amidst major policy changes during our observation period. Based on increasing regulations on e-cigarette products and the industry coinciding with the COVID-19 pandemic, we hypothesized that vape shop owners and employees would be more guarded and hesitant about participating in research, documented through changes in our vape shop recruitment efforts and related challenges. We also describe the systematic fluctuations and trends in: 1) e-cigarette product preference; 2) e-liquid nicotine concentration in mg/mL; and 3) frequency of use among vape shop customers during our observation period. We summarize the patterns in e-cigarette product use in a prospective manner to demonstrate that specific types of products would gain in popularity while others become nearly obsolete. In addition, we contextualize our findings with quotes during our recruitment process with vape shop owners/employees, interviews with customers, and with the product photos taken as part of data collection.

## METHODS

### Sample and data collection

All data were collected in the Southern California region from April 2019 through February 2023, and the sample consisted of 572 customer interviews within 83 vape shops, 11 of which required multiple visits to enable data collection from a sufficient number of customers available at the time of the visit. All interactions with the vape shop retailers were thoroughly documented, including the multiple attempts to obtain shop-level consent. Shop employees, managers, or owners were approached by data collectors and given information about the research study. They provided shop-level consent for data collectors to be in their shop location and approach customers as they exited the vape shop after making a purchase. Customers provided verbal consent and participated in a 15-minute structured intercept interview survey and were provided with a $35 gift card as participation compensation upon completion. The study was approved by the research institution’s Institutional Review Board (#HS-18-00732). In-person data collection paused during surges of COVID-19 infection and/or pandemic-related restrictions from April 2020 to November 2021. In order to protect privacy of recruited vape shops, all store names have been anonymized.

### Measures

*Vape shop level measures*

We maintained detailed records of our consent attempts at each candidate vape shop and noted reasons for refusal, if provided. We recorded the number of vape shops that we approached to obtain the shop-level consent and those that provided us with consent. All qualitative shop-based data were collected by documenting detailed notes directly after each consent attempt and/or visit to the shop. We also documented artifacts (e.g. posted flyers and their contents) at vape shops that were temporarily or permanently closed during or following COVID-19. We also report the content of any spontaneous communication with vape shop owners or employees in verbatim in the Results.

*Vape shop customer level measures*

The most frequently used type of vaping device (in the past 30-days) was assessed with the question: ‘What type of e-cigarette device do you use most often?’ [Response options: pen, box mod, disposable, pod mod (salt-based), pod mod (free-based), other]. Past 30-day vaping frequency was assessed with the question: ‘In the past 30 days, on how many days did you use e-cigarettes?’ (1–30 days). Preferred device/e-liquid nicotine level was assessed with the open-ended item: ‘How many mg per mL of nicotine does your favorite brand/flavor have?’ (e.g. 0, 3, 6, 50, etc.).

### Data analysis

We aggregated data by each month and treated each month as the unit of analysis. We employed a repeated cross-sectional design to document descriptive trends in shop-level recruitment efforts and vape shop customer-level variables during the observation period. Due to pragmatic and logistical reasons – including COVID-19-related restrictions – posed during the data collection period, there were a very small number of vape shops that were approached and agreed to participate for a large number of observational monthly-periods. Thus, our data structure does not render itself to a formal time-series analysis. With our method, we summarized the trend in the outcomes of interest instead, rather than to make an inference to the vape shop population.

## RESULTS

### Vape shop recruitment effort and success

As shown in Figures 2A and 2B, the number of shops our data collection team approached to obtain consent had to be substantially increased following the COVID-19 pandemic, peaking around March–April 2022 (about 40 shops), tapering off toward the end of 2022. To maintain comparable levels of shop-level consent rate, our research team was required to approach nearly twice as many vape shops, as represented by the red line which peaked during the first 6 months of 2022 (Figure 2A). The average monthly shop consent rate was 52.9% (SD=8.7), with an overall decreasing trend following the COVID-19 pandemic and changes in policy (except the end of 2022). In November 2022, our recruitment effort was concluding, therefore, we approached only 3 stores, all of whom consented (see the trendline Figure 2B).

**Figure 2:**
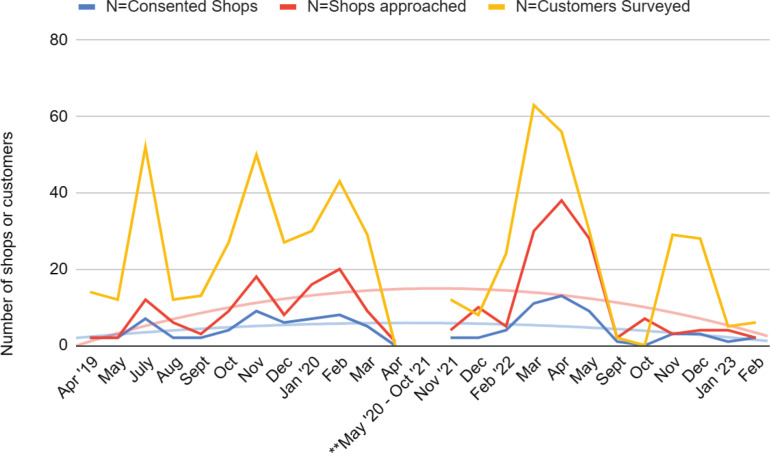
A) Changes in recruitment effort and success

**Figure 2:**
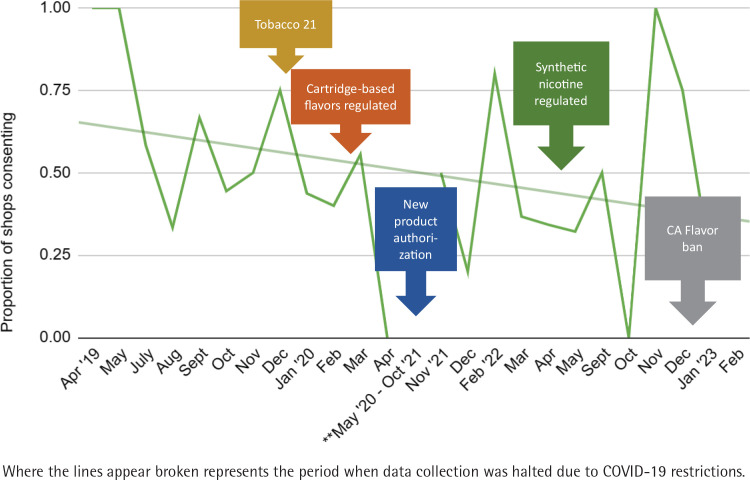
B) Shop-level consenting trend and policies

Our field notes corroborate the fluctuations in the receptiveness among vape shop owners or employees as policy changes unfolded. Examples of artifacts or refusal responses that our team encountered which align with the shop-level consent rate are listed below:

1. Flyer on door read: *‘[Shop] will be permanently closed due to the recent regulations that have passed*.’ (Oct. 2019)
2. Flyer on the front door mentions localized policy: ‘*[City] Ordinance No. 19-1940. An ordinance of the city council … prohibits the retail sale of electronic cigarettes, retail sale of flavored tobacco products and other vaping devices. Please help us stay in business … Only tobacco flavors are for sale*.’ (Feb. 2020)
3. Employee implies that customers have decreased due to localized policies, and the shop has been ‘*already affected by a vape ban*’. (Mar. 2020)
4. Employee stated that the ‘*business has been extremely slow*’. (Dec. 2021)
5. Employee stated that there is ‘*already too much going on*’, referring to the California flavor ban – SB 793. (Jan. 2023)

### Customer recruitment and success

We approached customers as they exited the store, attempting to recruit all customers (n=1012) present during the period of our presence in the shop. Eligible customers – those who had used vaping products in the past 30 days – were invited to participate in a 15-minute interview. Data collectors used scripts to verbally administer structured questionnaire items. Out of 946 eligible customers, 572 (60.5%) agreed to participate and completed the survey. The 66 customers who did not meet the eligibility criteria were excluded. The primary reasons for declining to participate in our study included: not having time (e.g. going back to their workplace, 42%), not being interested (41%), and not speaking English language (2%).

### Relative e-cigarette product categories purchased over time

**Figure 3:**
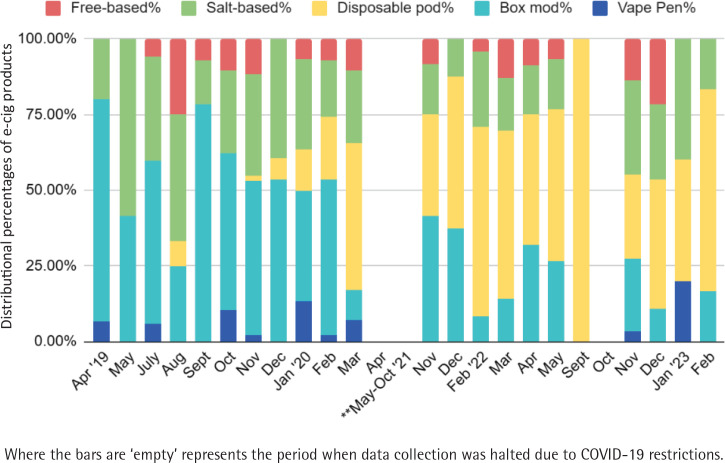
Distributional changes in e-cigarette product categories

We observed distinct changes in the distribution of e-cigarette products purchased at vape shops from 2019 to 2023. As can be seen in Figure 3, the distributional pattern demonstrates that the predominately used products changed with time. Specifically, box mod products (represented by light blue bars) were most popular in early 2019 and began to dwindle down as closed-system disposable products (yellow bars) became popular around March 2020, and continued their dominance through the end of the project observation period.

Salt-based products (green bars) were used at somewhat consistent rates throughout the project period and were primarily found in disposable and pod devices. Vape pens (deep blue bars), which constituted a small proportion of products used, were scattered throughout, and appear to peak in January 2023. Free-based products (in red) also make up a low proportion but were used somewhat consistently throughout the project period (Figure 3).

We also observed changes in types and intensity of the nicotine content within disposable vaping devices over the years. As seen in the photos of disposable products purchased by vape shop customer participants, more recent disposable products include synthetic nicotine with a warning label that emphasized ‘tobacco-free nicotine’ (Supplementary file Figure; all the photos taken in 2022, before the flavor ban went into effect in California).

### Changes in nicotine levels and use frequency

**Figure 4:**
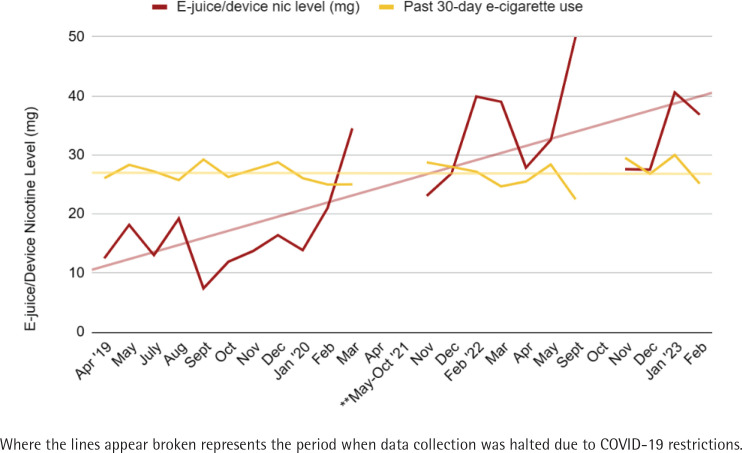
Changes in nicotine level and 30-day use over time

The aggregated data corroborates our field observation in which the average nicotine level in mg/mL (shown as a dark red line in Figure 4) started to increase substantially in February 2020 (39.9 mg/mL from 12.5 mg/mL in April 2019) with the upward trend continuing throughout the remainder of the project period (40.6 mg/mL in January 2023). This correlates directly with the dramatic increase in the purchase of disposable pod devices, as noted in Figure 3. However, despite the increase in nicotine level contained in products, the reported average days used in the past 30-day stays relatively unchanged over the project period (indicating nearly daily use, shown in gold line).

## DISCUSSION

In this study, we summarized various changes observed at vape shops and e-cigarette use characteristics over a span of 4 years, as policies and regulations have become increasingly restrictive affecting the e-cigarette marketplace. Specifically, we documented the challenges in data collection with vape shop retailers in Southern California, reflected by our research team’s shop-level recruitment strategies and efforts in relation to major federal and state policies implemented from 2019 to 2023. We also described the systematic fluctuations and trends in vape shop customers’ e-cigarette product preferences and nicotine contents over the same period. Given the constant evolution of vaping devices and e-cigarette characteristics, it is critical that regulatory agencies are informed about the latest trends in the e-cigarette market and the contributing factors associated with these changes. Changes to tobacco product characteristics can be a result of tobacco manufacturers’ attempt at complying with new policy regulations^28^. Monitoring and surveillance of emerging product characteristics and trends help policymakers and regulatory authorities to identify areas of priority for enforcement, as well as consider refinements in future regulatory efforts. A recent review of 104 studies published in 2022 concluded that high variety of flavors and high nicotine level concentrations may lead to higher abuse potential and appeal of e-cigarettes among users^29^. Further, e-cigarette flavors have been linked with increased use frequency among youth and adolescents^30,31^. Similarly, nicotine concentration levels – potentially bidirectionally related to e-cigarette dependence – could prove to be a highly concerning yet regulatable factor to curb nicotine addiction among established adult users.

Our findings merit further investigation of changes in the e-cigarette marketplace. With the variety and pace of changes in e-cigarette characteristics, future researchers need to examine how changes in e-cigarette characteristics affect nicotine level intake and use frequency, nicotine dependence, and harm perceptions among vape shop customers over time. This study provides insight into the developments of e-cigarette product preferences and behavioral changes among a large cohort of vape shop customers. The once most popular box mod e-cigarettes became rarely-purchased products by early 2020, around when disposable products began steadily gaining popularity through early 2023.

The social and policy climate has shifted in recent years and has contributed to changes within the e-cigarette product landscape. This important change in cultural climate was also illustrated by the changes in recruitment efforts as we report in this study. This is most likely due to stricter and more prevalent changes in tobacco policies in both the federal (e.g. FDA deeming rule) and localized levels of enforcement (e.g. city and/or statewide flavor bans), which were corroborated by our recruitment data. Recruitment effort had to increase, which was prevalent in all research since the pandemic. Our team attempted to contact a substantially greater number of shops (not needed in the past) and experienced additional challenges as new policies were introduced and implemented. Shops were concerned about going out of business due to sales restrictions and were reluctant to take on other activities such as being involved in a university-based research project. The current study could describe possible current and future challenges conducting research with tobacco retailers, even though such research is very much needed to further protect communities. Partnering with communities and the retailers in socially and economically disadvantaged areas might help to patch the disconnect that may exist between policymakers, researchers, and the vaping community. The partnership between our team and the vape shop retailer community enabled us to complete data collection as proposed, despite the challenges described in the current study.

### Limitations

This study has several limitations. Our study design and available data structure do not allow statistical inferences, as the same set of vape shops were not followed over time and we had small vape shop sample sizes per aggregation period used to summarize the trends. Further, our findings might not be generalizable to vape shops in regions outside Southern California, as well as e-cigarette users who purchase their e-cigarette products online or through other types of brick-and-mortar retail outlets, including youth (aged <18 years) and those outside Southern California. Given the nature of the self-reported customer data, the findings may have been influenced by recall and social desirability biases. As new regulations and policies were introduced, shop-level recruitment was affected as some shops were hesitant to provide consent to allow recruitment at their shops or share additional information about reasons for declining to participate with researchers, leading to potential selection bias.

### Implications

As product characteristics evolve and nicotine concentrations rapidly and continually increase in vaping products, it is important to consider stricter and more thorough regulations to circumvent such changes. This study provides documentation over time of the drastic increases in nicotine concentration among e-cigarette users as a result of the fluctuations in the product market. Regulating the nicotine content in tobacco products could provide a safeguard for future fluxes among e-cigarettes and other types of tobacco products. These measures may limit desirability among new users of e-cigarette products and protect future generations of potential users.

## CONCLUSIONS

The US FDA’s regulation of e-cigarette products has greatly changed the way the e-cigarette market operates and the availability of novel e-cigarette products available to users. Recent flavor restrictions of e-cigarette products warrant additional research to examine the changing practices of e-cigarette manufacturers and retailers post flavor-ban restrictions. It is important to continue surveillance of tobacco retailers to learn about new products that may be sold after flavor restriction policies are enacted. The US FDA recently announced plans for a proposed rule to establish a maximum level of nicotine in cigarettes, which may be applied to other tobacco products^32^. This will help policymakers establish additional regulatory policies to reduce the sales of new unauthorized tobacco products.

## Supplementary Material



## Data Availability

Monthly-aggregated data supporting this research are available from the authors on reasonable request.
