# Supplementary material for: Evaluating trends in recruitment challenges in vape shop research, e-cigarette product characteristics and use among shop customers from 2019 to 2023: A mixed-methods study
Source: Tob Induc Dis. 2024 May 23;22:10.18332/tid/175729. doi: 10.18332/tid/175729 (PMC11113008; doi:10.18332/tid/175729)
Supplement: Supplementary file 1 [file TID-22-86-s1.pdf]

Supplementary Figure. Photos of customer purchases of new flavored disposable vaping devices (containing 5% or 50 mg/ml of nicotine). Notice that disposable devices (B) and (C) contain "tobacco-free" or synthetic nicotine descriptions on the federal warning label. Also, (C) is an example of a disposable device with recharging capabilities.

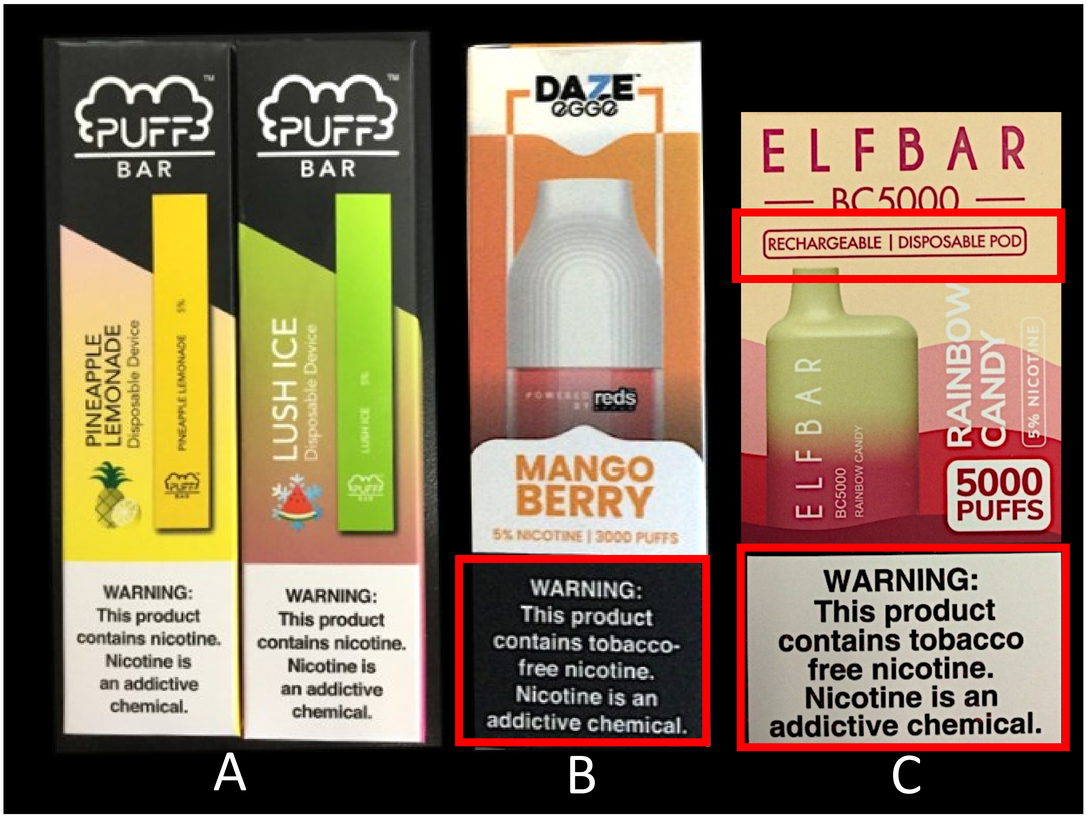

© 2024 Huh J. et al.
